# The Use of Biomaterials in Three-Dimensional Culturing of Cancer Cells

**DOI:** 10.3390/cimb45020073

**Published:** 2023-01-30

**Authors:** Novia Hanasti, Lia Faridah, Azzania Fibriani, Hesti Lina Wiraswati, Diah Kusumawaty, Savira Ekawardhani

**Affiliations:** 1 Department of Biotechnology, Faculty of Graduate School, University of Padjadjaran, Bandung 54211, SSWJ, Indonesia; 2 Department of Biomedical Sciences, Parasitology Division, Faculty of Medicine, University of Padjadjaran, Bandung 54211, SSWJ, Indonesia; 3 HSE Laboratory, KST UNPAD, University of Padjadjaran, Bandung 54211, SSWJ, Indonesia; 4 Department of School Life Sciences & Technology, Bandung Institute of Technology, Bandung 54211, SSWJ, Indonesia; 5 Department of Biology Education, Faculty of Mathematics and Science Education, The Education University, Bandung 54211, SSWJ, Indonesia

**Keywords:** 3D cell culture, biomaterial, cancer cells

## Abstract

Cell culture is an important tool in biological research. Most studies use 2D cell culture, but cells grown in 2D cell culture have drawbacks, including limited cell and cell-extracellular matrix interactions, which make it inaccurate to model conditions in vivo. Anticancer drug screening is an important research and development process for developing new drugs. As an experiment to mimic the cancer environment in vivo, several studies have been carried out on 3-dimensional (3D) cell cultures with added biomaterials. The use of hydrogel in 3D culture cells is currently developing. The type of hydrogel used might influence cell morphology, viability, and drug screening outcome. Therefore, this review discusses 3D cell culture research regarding the addition of biomaterials.

## 1. Introduction

Cell culture in biological research plays an essential role in understanding molecular mechanisms, the formation of tissues and organs, and disease-related status, especially in the mechanism of cancer development [1]. Cell culture is an extensively used technique in the drug discovery process for its simple, fast, and cost-effective way of working to reduce animal testing [2].

Most of the current research on cells is based on the two-dimensional (2D) cell culture. Two-dimensional cells have disadvantages regarding the limited cell-cell interactions and the extracellular matrix (ECM). Two-dimensional cell culture and its use in animal laboratories are considered the gold standard for modeling in cancer research. However, even though 2D culture has advantages such as its simplicity and cost-effectiveness when applied to investigate the behavior of cancer cells, 2D culture fails to accurately mimic the original cancer tissue’s complexity, as it does not recapitulate biological, chemical, and mechanical means [3]. Two-dimensional culture in drug screening often produces inaccurate results in modeling cancer cells due to its less heterogeneity, different phenotypes, and ECM structure, resulting in drug resistance seen in vivo [4].

The weaknesses of 2D cell culture can be overcome using in vivo modeling. However, modeling in animals still often cannot describe the actual situation like in humans. Compared to the 2D cell culture, cells grown in 3D culture can show changes to metabolic characteristics, such as increased glycolysis [5] and other interactions at the biochemical level and in the expression of complex genes and proteins [6]. Cells grown in 3D cell culture generally show decreased sensitivity to anticancer drugs [7]. Three-dimensional cell culture can describe some of the complexities of cancer cells that affect related biological responses, including drug resistance, invasion of cells, metastasis, migration of cells [8], and evaluating efficacy and pharmacokinetics in anticancer drug networks [9]. Three-dimensional cell culture can provide clear results regarding the effectiveness of anticancer drugs in drug screening [3]. In addition, 3D cell culture conditions can also mimic stroma-mediated effects on tumor development and drug resistance [10].

Cancer results from the abnormal growth of cells that are outside their normal limits. In pathological conditions such as cancer, the state of the cellular microenvironment also has an important role. Tumorigenesis and progression in cancer are not the only autonomous processes of this tumor; there are other influences, namely the surrounding microenvironment [11]. Cell interactions with this microenvironment provide an important influence on the impact of controlling anticancer drug responses [12]. Three-dimensional cell culture technology in drug discovery has been developed, including multicellular spheroids, on-chip organs, scaffolds, hydrogels, and 3D bioprinting [13]. In cancer research, the 3D cell culture model that is often used is the multicellular spheroid, in which cells will be collected and will multiply when cells are embedded in hydrogel [14]. The environment in 3D cell cultures better mimics the original environment likely to be experienced by cancer cells, whereby cell-matrix and cell-cell interactions can enhance the survival of these cells [8].

There are two methods to produce multicellular spheroids, namely, using a scaffold or not using a scaffold. By not using a scaffold or scaffold-free, physical intervention is used to force cells into forming a 3D structure, whereas by using a scaffold, cells will be able to grow into biomaterials [15]. Interactions with biomaterials will enable cells to enhance their proliferation, biological function, and differentiation, leading to the realization of interactions with the cancer cell environment. Biomaterials in 3D hydrogel cultures that have been widely used are the hydrogel poly N-isopropyl acrylamide-based microwell arrays (PHMA) studies [15], Matrigel™ dan PuraMatrix™ [16], PEO-GelMA [17], hydrogel agarose–collagen (A-C) [18], Poly(2-hydro2-hydroxyethyl methacrylate-HEMA) [19], polyethylene terephthalate (PET) [20], PEG-Maleimide (PEG-MAL) [21], poly(lactic-co-glycolic) acid (PLGA) or polycaprolactone (PCL) [22], polydimethylsiloxane (PDMS) dan gelatin methacryloyl (GelMA) [23]. Currently, several studies related to the addition of hydrogel in 3D culture cells are developing. Their developments are mostly in the application of various types of cancer cells and also with various types of hydrogels used. Cell morphology, cell viability, and drug screening are also influenced by the properties of each type of hydrogel used. The purpose of this literature review is to state the current developments in the use of natural, synthetic, or composite (mixed) hydrogels for 3D cancer cell culturing.

## 2. Hydrogels

A hydrogel is a 3D polymer network with cross-links that is capable of absorbing water or biological fluids even under pressure. Hydrogels have a high degree of hydrophilicity but is insoluble in water. A hydrogel can absorb as much as its dry weight in water or biological fluids because it has hydrophilic characteristics [24] and can maintain its network structures in three dimensions, as well as its polymer chain networks, in that condition. The ways hydrogel interacts with water or biological fluids are through capillary force, hydration strength, and penetration strength [25,26].

Hydrogels can mimic the natural microenvironment of cells. This makes hydrogel one of the most common tissue scaffolds [27,28]. Other properties possessed by hydrogel include, for example, hydrophilicity and fairly high water content; this is what makes hydrogels have a role in drug delivery [29]. There are several types of hydrogels. Namely natural, synthetic, and composite/hybrid (a mixture of natural-natural, synthetic-synthetic, and natural-synthetic). Natural hydrogels include gelatin, collagen, agarose and alginate, and the like Natural hydrogel is often used because it is non-toxic, easy to find, relatively inexpensive, and has biocompatibility and biodegradability properties [30]. This type of natural hydrogel has several drawbacks; for example, there are group differences in structure and performance, mechanical properties that tend to be unfavorable, and the potential for immunogenicity, which can cause limitations in the application of natural hydrogels [31,32].

**Figure 1:**
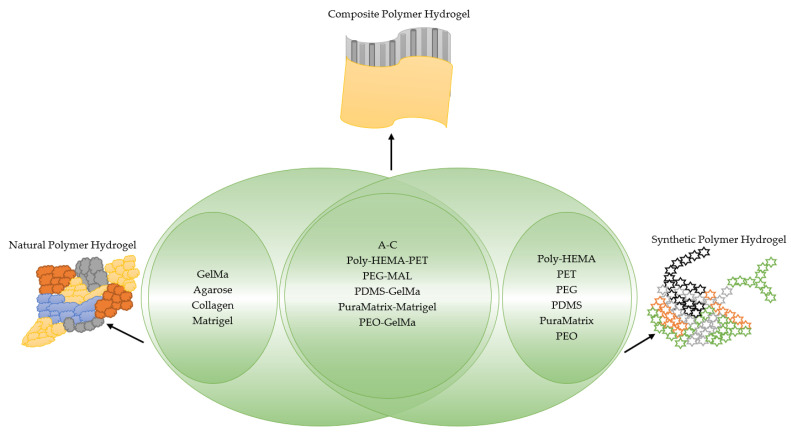
General polymer hydrogels developed for cancer cell culturing.

As for synthetic hydrogels, they include Poly(N-isopropyl acrylamide) (PNIPAAm), Poly(ethylene glycol) (PEG), alias poly(ethylene oxide) (PEO), PEG-Maleimide (PEG-MAL), Poly(ethylene glycol) (PEG), polyethylene terephthalate (PET), Poly-Lactic-co-Glicolyc Acid (PLGA), poly N-isopropyl acrylamide-based hydrogel microwell array (PHMA), and the like. Synthetic hydrogels have advantages when compared to natural hydrogels; for example, synthetic hydrogels can process polymers that use UV light in the polymerization process, have adjustable mechanical properties, and so on [33]. Synthetic hydrogel is also used in tissue engineering because this it has chemical properties and can be controlled in the production process [34]. Furthermore, the types of polymer hydrogel used for cancer cells are shown in Figure 1.

## 3. Natural Polymer Hydrogels

By using physical, chemical, or radiation cross-linking, the natural polymer hydrogels can be made from natural polymers such as cellulose, chitosan, gelatin, sodium alginate, dextran, and hyaluronic acid. Below are the most often used ones in 3D cell cultures.

### 3.1. Gelatin Methacrylate (GelMa)

Gelatin-based hydrogels are formed by cross-linking between network disulfides through oxidation due to the presence of hydrogen peroxide and physical interactions between the gelatin chains [35]. Gelatin methacrylate (GelMA) has an advantage in the 3D-bioprinting method; this is because this type of polymer has a high galvanizing speed at low temperatures and also adjustable mechanical strength. GelMA was synthesized by a direct reaction between gelatin and MA in a phosphate buffer at pH 7.4 and 50 °C [36]. Gelatin can form hydrogel physically at temperatures below 27 °C; if it is formed at body temperature, it will not be stable if you want to use it as a biomaterial because it is reversible thermal gelation, but this can be overcome, for example, by various chemical modifications and covalent cross-linking. Another thing is that this type of polymer can provide strong cell adhesion so that it can increase the interaction between cells and the supporting matrix (scaffold). Compared to, for example, collagen, gelatin has the advantage of better solubility and less antigenicity [37].

### 3.2. Agarose

Agarose hydrogel can dissolve in water at temperatures above 650 °C and can form gels at temperatures ranging from 170 °C to 400 °C; this depends on molecular weight and chemical modifications [38]. The mechanism of agarose gelling lies in the intra- and inter-molecular hydrogen bonds under the cold conditions, which can lead to aggregation of the double helix through physical bonds in the molecule [39]. The level of viscosity and elasticity of the agarose hydrogel depends on the concentration of the solution and the resulting molecular weight [40]. Agarose has been widely used for biomedical applications because it has properties in controlled gel formation, is water-soluble, has the adjustment of mechanical properties, and is non-immunogenic. Based on the level of stiffness and functional groups, agarose can support cell adhesion and proliferation [41]. In research, agarose hydrogel has been widely used, for example, in cancer therapy [42,43] and tissue engineering applications such as of the nervous system [44] and bones [45].

### 3.3. Alginate

Alginate is a type of polymer that has the characteristics of biodegradability, biocompatibility, non-immunogenicity, and non-toxicity [46,47]. The components present in alginate are brown algae cell wall components and exopolysaccharides from Pseudomonas and Azotobacter bacteria, which are applied in various industries such as textiles, food, and pharmaceuticals [48]. Alginate contains many hydroxyl and carboxyl groups; this allows alginate to form intermolecular hydrogen bonds. The physicochemical properties of alginate are very dependent on the composition of the monomer sequence, functional groups, and molecular weight [49]. Alginate cano forms hydrogels through ionic cross-links, which make it easy to accept cell encapsulation. In a 3D cell culture system, the negative salt charge possessed by alginate allows the formation of a reproductive hydrogel [50]. Alginate can also be used in modeling radiobiological responses and metabolism in cancer cells, as well as in cell transplantation [51,52]. In contrast to collagen, alginate must be modified with adhesive ligands, such as the tri-amino acid sequence arginine-glycine-aspartate (RGD) peptides, to activate cell attachment [53].

### 3.4. Collagen

Collagen is considered one of the most important biomaterials in regenerating connective tissue because this collagen has hydrophilicity, good biocompatibility, low antigenicity, flexibility, and excellent biodegradability [54]. The main advantage of collagen is its biomimetic properties; besides that, it is also cytocompatible, that is, it can be accepted by adhesion cells without modification. The weaknesses of collagen itself are, namely, the low level of stiffness and limited stability in the long term [55,56]. Collagen is usually soluble in acidic solutions and can be processed into various forms, such as powders, sponges, and foams. Collagen is the most common component of the ECM and can maintain the integrity of its biological structure. This is what makes collagen able to create a microenvironment from ECM mimetics which can be beneficial for knowing various cell functions, for example, adhesion, proliferation, differentiation, and so on [57]. Collagen hydrogel consists mostly of several types of collagen, namely: Collagen type I, Collagen type II, and Collagen type III. Type I collagen is the most common type and is a major component of many tissues.

## 4. Synthetic Polymer Hydrogel

### 4.1. Poly(N-Isopropyl Acrylamide) (PNIPAm)

Poly(N-isopropyl acrylamide) (PNIPAm) is a thermosensitive polymer that exhibits LCST and is frequently used for cell culture, drug delivery, and gene delivery systems [58,59]. PNIPAm has a low intrinsic value (lower critical solution temperature) at 320 °C, where reversible hydrophilic-to-hydrophobic transport occurs [60]; moreover, the hydrophilic nature below that of LCST provides facilities for cell release. The way for this thermoresponsive cell release to occur is to avoid damage to the cell membrane and maintain cell-to-cell integrity without removing ECM protein [61].

### 4.2. Poly(Ethylene Glycol) (PEG)

Poly(ethylene glycol) (PEG) has characteristics that can be used in 3D cell culture applications in the form of mechanical properties that can be adjusted to flexibility, resistance to intrinsic protein adsorption, and adhesion to cells [62]. Poly(ethylene glycol) (PEG) is also used in smooth muscle cell (SMC) proliferation in 3D cell culture [63]. Poly(ethylene glycol) (PEG) is one of the biocompatible synthetic polymers; it is also the most frequently used in the biomedical field, derived from materials that are hydrophilic and cannot be decomposed hydrolytically. It has a high degree of water solubility, and there are many organic solvents. PEG also has properties that are not too high in toxicity [64]. PEG can be used in microbiology on aortic valve interstitial cells (AVIC) [65].

### 4.3. Polyethylene Terephthalate (PET)

Polyethylene terephthalate (PET), namely polymer terephthalic acid (TPA) and ethylene glycol (EG). Because it induces only a small degree of an inflammatory response, PET can be used as a biomaterial [66]. It has advantages, including characteristics which are biocompatibility, rigidity, hardness, high thermal properties, good insulation, and resistance to corrosion from chemicals [67,68]. In addition to the advantages of PET, it also has disadvantages, for example, attachment to cells and low compatibility; this is because PET has a hydrophobic surface that is not chemically reactive (inert) [69]. PET is often used as a material for technical and medical applications, for example, in developing a laser method for drilling holes in PET to obtain a micro-sieve that can capture prostate cancer cells, testing adhesion to Escherichia coli bacteria [70,71].

### 4.4. Poly-Lactic-co-Glicolyc Acid (PLGA)

Poly-Lactic-co-Glicolyc Acid (PLGA) is a synthetic copolymer that is often used to increase activity against anticancer drugs because it is biodegradable and can be used for drug delivery, it is biocompatible, non-immunogenic, has good stability in preparation for storage, and is not toxic [72,73]. PLGA has been approved by the United States Food and Drug Administration and European Medicine Agency (EMA) and can be used for drug delivery in humans [15]. PLGA is often used as a biodegradable polymer because PLGA undergoes hydrolysis in the body to produce biodegradable and biocompatible metabolic monomers, namely lactic acid and glycolic acid.

## 5. Applications

To date, the extensive applications found of the various biomaterials in the 3D cell cultures for the cell lines derived from the cancer cells are summarized in Table 1 and then explained in more detail according to the type of cancer cells. We found that breast cancer and lung cancer were mostly investigated in the studies employing 3D cancer cell culturing.

### 5.1. Breast Cancer

#### 5.1.1. Drug Sensitivity

The studies mainly used MCF-7 cells and cisplatin [18,19,20] in the addition of different types of hydrogels such as agarose–collagen (A–C) [18], hydrogel polyethylene terephthalate (PET) [20], or Poly(2-hydroxyethyl methacrylate) (poly-HEMA) [19]. Faster diffusion results in a softer hydrogel, reaching 100% after incubation for 2 h at 37 °C. The concentration of cisplatin used in those studies was 100 µm. DNA damage induced by cisplatin did not decrease when the 3D-reconstituted basement membrane (3D-rBM) method was applied [19]. A slot blot test for immunodetection of the addition of cisplatin-DNA was also carried out [19] and showed that the level of DNA damage in cells grown in that 3D culture was not reduced. It was concluded that the cells grown in this condition were more resistant to cisplatin and got persistent DNA damage.

The study using polyethylene terephthalate (PET) hydrogel for a 3D co-culture model showed that PET-based cell cultures can cause drug resistance to tamoxifen in the MCF-7 cell line [76].

In the study using PEG-Maleimide (PEG-MAL) hydrogel for 3D multicellular tumor spheroids (MCTS) using AU565 and BT549 cell line [21], when the drugs used were cisplatin or paclitaxel as the inhibitors of Raf Kinase [77], the results showed that drug resistance was quite high in this model.

Polydimethylsiloxane (PDMS) is often used because it is more flexible, has biocompatibility, transparency, and also low-temperature drying [78]. In the study using a pseudo-3D culture method, doxorubicin (DOX) was still potent to the cancer cells.

Methacryloyl gelatin (GelMA) has a modulus that is almost the same as that in breast tumors [79]. MCF-7 cell line grown in GelMA showed lower viability when compared to the ones grown in ordinary substrates upon treatment with DOX because most of the cells detached from the surface [23].

In the model using the 3D microfluidic chip method and matrigel with the addition of carbonic acid and DOX for the cell lines MCF-7, MDA-MB231, and MCF-10A, carbonic acid was shown to be more cytotoxic against the MDA-MB 231 cell line, while DOX was more effective against the MCF-7 cell line [74].

#### 5.1.2. Cell Morphology

One of the main advantages of hydrogels is that they can produce results that are as realistic as in vitro conditions. The appearance of spheroids in the stiffer hydrogel type is more spherical in shape but smaller in size when compared to the conditions in the softer hydrogel type. MCF-7 and MDA-MB-361 cells were able to form spheroids, while MFA-MB-231 cells were unable to form spheroids in a certain hydrogel. The stiffness of the hydrogel type and the composition of the matrix will regulate the growth and morphology that will be produced [18]. For example, the morphology of the MCF-7 cell in poly-HEMA with the 3D-RBM method was the formation of a ball-like structure [19]. The number of cell ratios in cultured cells may have a significant change or impact on changes in morphology, cell-to-cell interactions, and also for resistance or sensitivity to cancer drugs [80]. The morphology of MCF-7 cells in PET hydrogel was a relatively small and well-organized after being planted for 72 h; at the same time, NIH-3T3 cells displayed a long structure and had fibers [20]. Cellular aggregates can significantly increase the interaction between cells and cells-ECM, therefore mimicking the shape and stiffness of more specific tissues [13,81]. In polyNIPAAM hydrogel, MCF-7 and BT474 cells formed the multicellular tumor spheroid (MCTS) structure, while SkBr3 and MDA-MB-468 cells were shaped like grapes [21].

#### 5.1.3. Viability

Cisplatin treatment on the MCF-7 cell grown with 3D-RBM still resulted in significant cell death [19], while the treatment of DOX in 3D-microfluidic chips with PDMS hydrogel showed resistance with cell viability only at 19.06% for MCF-7, at 20.80% for MCF-10A cells, and at 37.50% for MDA-MB 231 cells. For the butterfly-shaped microchips using matrigel with the addition of DOX, the respective cell viability was at 41.41% for MDA-MB 231 cells, at 36.28% for MCF-7 cells, and at 22.40% for the MCF-10A cells. After the addition of carbonic acid, cell viability was at 31.47% for MDA-MB 231 cells, MCF-7 cells at 40.76%, and MCF-10A cells at 14.94%. Generally, cell viability will be higher in microchips than with 2D and 3D cell culture systems due to the resistance generated by the cells to the applied drug [74].

MDA-MB-361 cell spheroids were still viable after 8 days of incubation in agarose hydrogel. After 2 weeks, the initial aging was seen on the 0.25% agarose while it was still stable in the agarose–collagen (A-C) hydrogel with a concentration of 0.125% to 0.02%. Spheroids that grew on a softer hydrogel could survive until they reached a larger average size; however, spheroids in an agarose–collagen (A-C) hydrogel stopped growing after 2 weeks and were totally dead at week 4. These results suggested the ability of a less rigid hydrogel to accommodate spheroids leading to slower senescence and cell death. [18]. The greater the amount of agarose, the stiffer the hydrogel produced. The larger the biomolecule and the stronger the type of non-covalent interactions formed by the matrix, the slower and the fewer the number of molecules that reached the cells embedded in the matrix. The hydrogel will act as a barrier for the diffusion transport of nutrients as well as certain drugs in the formation of spheroids [82].

### 5.2. Lung Cancer

#### 5.2.1. Drug Sensitivity

The cell lines used in reviewed studies were A549, MG-63 Hela, and Human Lung Fibroblast (HLF) [16,17,75], and the hydrogel used was Poly N-isopropyl acrylamide-based hydrogel microwell array (PHMA). PHMA has thermoresponsive properties, allowing the attachment and growth of cell aggregates or spheroids at 37 °C. MG-63 spheroids are relatively intact and round with tightly arranged cell shapes, which makes it easier to visualize changes in spheroid morphology to drug response. DOX exposure to MG-63 spheroids showed concentration-dependent cell death with slight resistance after 72 h incubation, i.e., a viability of 39.2 ± 7.4% for 1 µM and 24.2 ± 2.9% for 10 µM treatment [75].

In the experiment using Matrigel™ and PuraMatrix™ for A549 cell and decotaxel treatment, the results showed that 3D A549 cells in 0.25% and 0.30% PuraMatrix™ had low chemoresistance to decotaxel.

In the experiment combining 3D culture with digital light processing (DLP) and PEO-GelMA to make porous microgels for A549 cell lines with paclitaxel treatment, the results showed a higher gene expression associated with the ROCK pathway induced by 3D culture and an increase of paclitaxel resistance in lung cancer cells. The environmentally-induced ROCK pathway of these cancer cells also plays an important role in the drug sensitivity of lung cancer cells present in this microgel environment [17].

#### 5.2.2. Cell Morphology

Cell morphology, gene and protein expression, cell polarity, viability, cell proliferation, and response to stimuli will be affected as a whole, depending on the environment created in either a 2D or 3D culture system [83]. Using different cell types (A549, HeLa, MG-63, and fibroblast cells) in PHMA showed that the morphology of HLF cells and MG-63 cells decreased slightly in diameter until day 5 for HLF cells; in contrast, MG-63 cells increased in diameter until day 7. For A549 cells, it takes a relatively long time to form spheroids [75]. This morphological difference can be attributed to differences in the adhesion properties of each cell line, one of which plays an important role in this difference is E-cadherin. In addition, morphological changes in 3D culture cells are associated with the Rho-associated kinase (ROCK) signaling pathway.

In the study [17], using porous microgels resulted from the combination of DLP and PEO-GelMA. The A549 cell grown in it showed unique morphology of the spherical form and similarly grew into multicellular spheroids.

When the same A549 cell line was cultured in Matrigel™, cell morphology had become that of a smooth cell surface, whereas in PuraMatrix™ the cell surface was rougher with protrusions (invadopodia) and membrane wrinkles that were different from cells grown in the other 3D cell culture technique. The expression of E-cadherin grown in Matrigel™ or PuraMatrix™ can only be seen at a concentration of 0.15% and not at the concentrations of 0.25% and 0.30%. It was also shown that the stiffness of the cell substrate or structure of the 3D culture was not related to the expression of Epithelial-to-Mesenchymal (EMT) protein markers [16].

#### 5.2.3. Viability

The use of PHMA for HeLa spheroids supported the growth of intact spheroids until day 7. Spheroids also showed an increased cell viability and proliferation until day 8, and then cells were saturated. With a cell density of 24 × 10 cells/pHMA, viability was observed until day 3, and yields remained constant; therefore, 24 × 10 cells/pHMA was considered the optimal cell seeding density [75].

In the experiment using the A549 cell line for 3D culture with PuraMatrix and Matrigel, it was found that 0.25% and 0.30% Puramatrix resulted in a decrease in cell viability for DOX treatment above 50 nM. The results from using the porous microgels of DLP and PEO-GelMA combination showed that the cells were still able to maintain good cellular activity with almost no dead cells [16,17].

## 6. Conclusions

In conclusion, various studies have carried out 3D cell culture systems with the addition of biomaterials. The addition of gels and scaffolds has several advantages in simulating the 3D structure of cancer cells in vivo when compared to other culture methods. The type of hydrogels used in 3D culturing could affect general cell viability, cell morphology, and cancer cell sensitivity to drug or chemoresistance. In addition, several biomaterials and cells may be combined according to their properties and functions to enable a 3D cell culture system that more closely resembles the realistic environment of in vivo cancer cells.

## Figures and Tables

**Table 1:** Three-dimensional cell culture of cancer cells combined with biomaterials.

Biomaterials	Drug Screening	Cell Line	Cancer Cells
Agarose–collagen (A–C)	Cisplatin 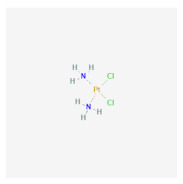	MCF-7, MDA-MB-361, and MDA-MB-231 [18]	Breast cancer
Poly(2-hydroxyethyl methacrylate) (poly-HEMA)	Cisplatin	MCF-7 [19]	Breast cancer
Polyethylene terephthalate (PET)	Cisplatin Tamoxifen 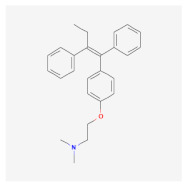 Oxaliplatin 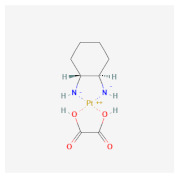	MCF-7 and mouse fibroblast NIH-3T3 [20]	Breast cancer
PEG-Maleimide (PEG-MAL)	Cisplatin Paclitaxel 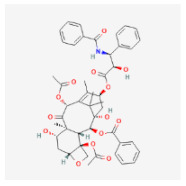 Sorafenib 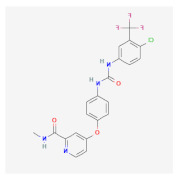 Mafosfamide 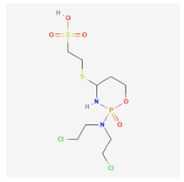	MCF-7, AU565, BT549, LNCaPcol, PC-3, SKOV-3, OVCAR-3, HCC 1419, HCC 1428, MDA-MB 231, SkBr3, ZR-75-1, BT474 [21]	Breast cancer, Prostate cancer, Ovarian cancer
Polydimethylsiloxane (PDMS) and gelatin methacryloyl (GelMA) hydrogel	doxorubicin (DOX) 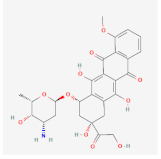	MCF7, SKBR3, dan ZR-75-1 [23]	Breast cancer
Polydimethylsiloxane (PDMS)	doxorubicin (DOX)	MCF-7 dan MDA-MB 231 [74]	Breast cancer
Poly-Lactic-co-Glicolyc Acid (PLGA)	4-hydroxytamoxifen (4-HT) 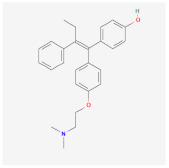	T47D [22]	Breast cancer
poly N-isopropyl acrylamide-based hydrogel microwell array (PHMA)	Doxorubicin (DOX)	A549, MG-63 Hela and Human Lung Fibroblast (HLF) [75]	Lung cancer
PuraMatrix™ and Matrigel™	Decotaxel 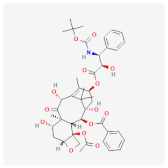	A549 [16]	Lung Cancer
Polyethylene oxide—Gelatin methacryloyl (PEO-GelMA)	Paclitaxel 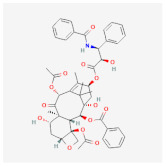	LL/2 cells (mouse Lewis lung carcinoma), A549 cells (human lung carcinoma) and NCI-H1975 cells (human lung adenocarcinoma) [17]	Lung Cancer

## Data Availability

Not applicable.
